# Are All Men, Men, or Are They Something Else?—Types of Mankind

**Published:** 1856-01

**Authors:** 


### REVIEW DEPARTMENT.

### ARTICLE VIII.

*Are all Men, Men, or are they something else?—Types of Mankind.* By J. C. Nott, M. D. Mobile & George Gliddon, Philadelphia, 1854.

It so happens that we have not sufficient matter of a truly professional character to occupy the pages of the present number of our journal, and we are, therefore, at liberty to bring before our readers the highly interesting and important topic discussed in the bulky volume above indicated, which has attracted much attention and passed through several editions.

The identity and common origin of the human family has, until recently, been very seldom called in question. Men did not dispute each other's title to manhood, however little they governed themselves by the kindly considerations which should be generated by consanguinity. Sometimes, to be sure, a restless speculation in things suggested that the Europeans were a little too familiar with other folks, and had been a little too fast in admitting Africans and Asiatics of certain hues and shapes, to the honors of technical fraternity, but the imitation was not regarded as worthy to create a serious suspicion of common humanity. People have long since been accustomed to hear foolish things from philosophers, and so they were contented to admit, theoretically, all races of men to the right of brotherhood, being only careful that none of these rights should be transmitted into practical proportions in good, or exemption from evil, further than it might be convenient for the dominant class to grant the one or remove the other.

Recently, however, there have been repeated and persevering attempts to unsettle the quiet of an opinion on this subject, and the huge volume, whose title we have quoted, is the result of a coalition of three scientific gentlemen, to affect, if possible, this very desirable end, that men, now not loving each other as brethren, may hereafter hate each other as brutes. Messrs. Nott & Gliddon are the manufacturers of the book. The late Dr. Morton, of Philadelphia, is made to contribute largely to it. Prof. Agassiz lends it the credit of his name, and encumbers it with a very presumptuous and shallow essay to prove the existence of some mysterious law, independent of climate or known determining causes, by which certain varieties of men, brutes and vegetable productions are marked off and associated together, on the face of the earth. Dr. Patterson contributes a biography of Dr. Morton, and lastly, but not least, Mrs. Gliddon furnishes the illustrative drawings, which we are compelled both by gallantry and truth, to pronounce the best part of the whole performance. Besides all these contributors to the material of the book, Mr. Gliddon acknowledges the assistance of several friends, who jointly and severally, afforded him important aid in putting what he had to write, into good possible English; a friendly act rendered necessary by a peculiarity of Mr. Gliddon, with whom, as he informs us, "style is ever subordinate to *matter*."

We cannot pretend to review this conglomerate in the few pages we can devote to it; we can only point out a few of its many errors and imperfections, and express our opinion of its design and character.

We do not know whether we ought to consider the work as one of physical science or theological controversy. The subject might certainly have been treated as one purely physical. The writers might have confined themselves simply to collecting and grouping facts, and reasoning calmly and closely from the phenomena before them, leaving their deductions to work out their own consequences as other truths, or to be exposed and cast aside as other errors. As they chose to avail themselves of records and traditions, they might have consulted the historical scriptures, and given their testimony such weight as they in their associate wisdom, should have determined. Messrs. Nott, Gliddon & Co., might have been respectful to the opinions and faith of the people for whom they wrote, and yet have presented all the treasures of their knowledge, as exhaustively and effectively as they did. They have chosen to do otherwise. They have preferred to make their work a formal attack upon the authenticity and authority of the bible. In their enthusiastic egotism they have made haste to announce their hostility to that venerable book, and they appear before the public rather as the champions of Deism than the expositors of nature.

As Mr. Gliddon has resided for a long time among the Turks, we are not surprised that he has learned some infidelity, especially, as notwithstanding his pretensions, he does not seem to have learned much else. If we cannot then excuse Mr. Gliddon, we can, at least, make allowance for the unfortunate circumstances of his life, but the other parties to this unholy alliance are inexcusable, if not for unbelief of christianity, yet for their hatred against it. As a sample of the temper with which Dr. Nott approaches a physical investigation, read the following paragraph.

"On former occasions we had attempted to conciliate sectarians and to reconcile the plain teachings of science to theological prejudices. In return, our opinions and motives have been misrepresented and vilified by self-constituted teachers of the christian religion. We have, in consequence, now done with all this, and no longer have any apologies to offer nor favors of lenient criticism to ask. The broad banner of science is herein nailed to the mast. Even in our brief day we have beheld one flimsy religious dogma after another consigned to oblivion, while science on the other hand, has been gaining strength and majesty with time." Mr. George Gliddon is equally warlike, while Dr. Nott "nails his colors" to the mast, and lets his saucy craft career freely over the ocean to the jeopardy and at the proper risk of any "flimsy dogma" that may venture across his bows. Mr. Gliddon announces his purpose to "carry the war into Africa," the land where he learned to fight. He avows his cruel resolution to pursue christianity "even to the death," and informs us at last, that besides the stabs he has administered by this volume, he has three hundred pages kept in reserve. These he calls his "powder," and tells us that he is "keeping it dry" for a final bombardment of christianity. If it shall prove "drier" than the pages he has already printed, the reading world is indeed threatened with no ordinary evil.

The design, at least one design of the book is, to controvert christianity by "science," in other words, to show that the teaching of revelation is contradicted by visible facts, and, therefore, cannot be true, and especially that the fundamental doctrine of the bible which regards mankind as a family, derived from one pair, Adam, and subsequently from the one family of Noah, is erroneous.

We will not dwell upon the important moral and religious bearings of the propositions stated by the collaborators of this volume; we are willing to consider the question of the identity of the human race as a purely physical one. If men are not of one blood; if they are descendants of progenitors originally and primordially distinct, if the distinction be perpetuated by definitive organization, then we will give up the bible, and adhere to

Mr. Gliddon. We only ask that the facts be clearly and satisfactorily made out. This done, and infidelity has triumphed; and the European race take the place of God in the earth.

We will pass over the essay of M. Agassiz. It is the assertion and defence of a mere conceit, and is scarcely worthy of examination. It is contradicted by every thing and every body, and even by M. Agassiz himself, for, in admitting the identity of the great American race, from the arctic circle to the equator, he virtually admits that his whole speculation is nonsense.

M. Agassiz having delivered his argument against the original unity of the race, and insinuated that men, so far from being cosmopolites, are assigned by few of original creation to certain geographical districts. Dr. Nott and Mr. Gliddon proceed to divest the public mind of the vulgar error, that men originally came from Asia, and to show that the notion of unity of races is a comparatively modern conceit, altogether absurd, never having been conceived by any primitive nation, such as Egypt or China. They think it was borrowed from the Bablyonians, or from the Buddhists, or somewhere else. *They do not find it in Genesis*, and if they did, they consider Moses very indifferent authority for anything. They would consider the sarcophagus of an Egyptian of Moses' day, unimpeachable evidence of anything they might spell or suppose themselves to spell out of the hieroglyphics on his mummy case, but they have no faith in any old writing more distinct and intelligible.

Dr. Nott asserts, that "There exists a genus Homo," embracing primordial types or species which have remained permanent and untransitional, through all recorded time, and despite the most opposite moral and physical influences. From this fact he argues that these species always were distinct, and that men were originally created in nations, separate and distinct from each other.

The argument for this bold assertion, not by any means modestly stated, rests mainly upon the antiquity of the Jewish and Negro lineaments, as affording types, least confused by mixture and best attested in antiquity. The Egyptian monuments furnish the pictorial testimony to the fact, that in the days of Egyptian lithoscripsy, the Jewish features were formed and recognized as typoidal of the Abrahamic family. From this it is inferred that these features *always* so existed, and that they are the result of primordial organization, constituting a specific mark for the Jewish creation.

To this we have several objections.

First, we cannot admit that the identity of the Jewish features are so clearly made out by the Egyptian monuments. The process of reasoning upon these Abrahamic portraits is rather too circular to come to an end. All the Jewish looking faces are first judged to be faces of Jews, and then the Jewish face is concluded to have been always peculiar to the descendants of Abraham. Now we will venture to assert that neither Dr. Nott nor Mr. Gliddon can distinguish a Jew with anything like accuracy. That there is a certain peculiarity of countenance common among the Jews is true, but it is not at all uncommon among gentiles, and when necessary to a theory it has been found equally prevalent among American Indians and Affghans. We know Americans who are much more like Jews than many of the Jews are, according to Dr. Nott and Mr. Gliddon, and we doubt not that many of our Saxon race would be referred to the Judaic creation, were their profiles examined by our "scientific" authors, with as much discrimination as those of Egyptian portraiture. Some of the kings and queens of Egypt present the features assumed to be Jewish; hence, without more ado, they are declared to be Jewish, and hencc the Jewish face is declared to have been peculiar to the Jews in the days of the royal personages presenting it. The very contrary is in fact the prima facia suggestion. We would not look for Jews on the Egyptian throne, and, therefore, we should infer that the countenance assumed to be peculiarly Jewish, was occasionally met with among the Egyptians, just as it now is among the Europeans and Asiatics, and, therefore, was then as now, not a peculiarity at all, though more common among the Jews than others. To say the least, this appearance of the Jewish face on the Egyptian monuments, should not be relied on as an argument for the typical import of it. But there are other difficulties about this Jewish face. The Jews are of comparatively very recent origin. They date only from Abraham, but we suppose that "science" does not pretend that the "nation" was then created. Abraham then was but one man of a nation, all having "Jewish" faces. It is to be supposed that if this one man propagated a nation, and diffused the face through them, other men of the nation did so too. An enormous multitude, then, among whom the Jews are but a handful, must have inherited the "face." How then did it become distinctive of Abraham's family? If all the rest lost the "face" by intermixture, then certainly this "type" of race has none of the "permanency" about it which marks species; again, had there been no intermixture before Abraham's day? And, if there had been, why may not the "face" be a result of mixture? Who shall say that it was the stamp of the original progenitors, and not an accidental inheritance from more recent ones? We can readily perceive that a bold outline of countenance might be impressed by a parent upon his immediate descendants, and that they by intermarriage might perpetuate the family likeness. We see that this does occur among the lower animals, and even in the human family it has been observed, as in that of the reigning house of Austria. We do not perceive why this family resemblance may not be diffused as widely as the family: as widely, at least, as it diffused among the Jews, a great number of whom are entirely indistinguishable from the people with whom they dwell. Be this as it may, it is certainly a violent conclusion that what we call national peculiarities of feature, are really specific marks of distinct human creations. The features of the Irish, Germans and Italians are quite as well defined as those of the Jews; yet we are loth to believe that these several people are indigenous to the countries where we find them. All these faces disappear in America in two generations and pass into another, from which some future Gliddon may satisfy the scientific world, that we were all indigenous to these lands, and that like Topsey, "we wasn't born,—we grow'd."

The negro face is of course brought prominently before us, to show the permanence of the type, and the impossibility of common origin.

This peculiar countenance is found upon the Egyptian monuments assumed to be of a date 2400 years before Christ, hence it is argued that the negro face always existed since the creation. This we protest against, as an outrageous non sequitar. It amounts but to a demonstration that negroes, whether many or few, were found in those days. We by no means admit that the Egyptian chronology, or the professed interpretation of it is correct. At most, the chronology of the monuments can claim to be only probable. Great blunders have been made, and no doubt are yet made with regard to the age of these monuments, but admitting with Bunser and Leipsius, that the old Egyptian monarchy dates at 3893 B. C., we have an interval of fifteen hundred years in which no negroes can be found. Mr. Gliddon admits into his work a *fact* and inference which proves the human race to be more than 57,000 years old. With what propriety then can he talk of the antiquity of the Egyptian monuments? These records are recent, absolutely of more modern date compared with the 50 or 100,000 years preceding them. Suppose negroes did exist a few days ago when the Pharaohs and Moses lived, what of that? We might as well argue that the hairy woman existed from eternity, because her likeness has been exhibited for a year or two, as to infer that because negroes existed four thousand years ago, therefor, they existed for interminable ages?

We opine that Mr. Gliddon knows very little about Egyptian antiquities, further than is sufficient to enable him to distinguish the sex of a mummy after it is unrolled. Bunser and Leipsius probably knew a great deal more, and they both are firm believers in the unity of our race. Had the Egyptian monuments showed any thing to the contrary, they would have told us so. Until they shall do so we must be excused from confiding in Mr. Gliddon.

The permanency of the North American Indians is shown by observations made at an excavation in New Orleans, when a skeleton of a man was discovered at the enormous depth of sixteen feet in the alluvial mud, the cranium being under the roots of a cyprus tree. From these data it is elaborately ciphered out that the skeleton aforesaid was alive upon the earth 57,000 years ago, and was then an American Indian! This information is furnished by Dr. Bennet Dowler, who has thus proved not only the longevity of the Indians but the indestructibility of their skulls, and in so doing furnished a much more *permanent* mark of specific difference between them and other folks than any before suggested. Well may a reviewer in the Southern Quarterly Observer, make "inferences" founded on a calculation so totally in conflict with the known progress of history and human development; with the mature convictions of Lyell, Murcheson and the soundest judging geologists; with the comparatively recent dates of the oldest recorded astronomical calculations; the most ancient of which ever heard of, Laplace tells us, in his System du Mordi, are some rude Chinese notices of eclipses 2000 years B. C., and the first that can be relied on at all only 1100 B. C., and with the limited range of even Egyptian chronology are too preposterous to require serious refutation.

If we observe the lower animals we find that every species, though carefully guarded by a natural law from being confounded with others by admixture, is capable of developing many, and under favoring circumstances, permanent varieties. The domestic animals are familiar witnesses to this fact. The hog in his wild state is more different from the hog domestic, both in shape of skull and other peculiarities than the Hottentot is from the Caucasian. Turn the domestic hog loose into the forests, and he returns to the original type. Wild dogs do not bark, the domestic dog turned loose, ceases to do so. Domestication begets a variety of colors which are lost when domestic animals become wild. The sheep becomes hairy in hot, and the horse woolly in cold, climates. Yet in all the changes observed in the lower animals we perceive nothing which invades the limits of species. The wild hog is as evidently a swine as the tame one; the wild horse as the tame one, and there is nothing in all that we observe of the occurrence and permanency of varieties among them to interfere with the opinion that originally they were from one pair.

With regard to mankind, it is evident that within the limits

5*

of species there is freedom of variety to a great extent, and that among them, as among other animals, varieties may become permanent. Black color is no longer considered peculiar to the African race. How it originated we cannot tell. We do not know the circumstances which in early ages affected the human family, nor how soon its tendency to develop permanent varieties may have reached its ultimate limits, and in some respects, perhaps been exhausted. "It follows from many facts," says Lyell, "that a short period of time is generally sufficient to effect nearly the whole change which an alteration of external circumstances can bring about in the habits of a species." Mr. Gliddon, however, has furnished us abundant time for any thing to be done which time can do, for he has given us at least 50,000 years, with the privilege of as much more if necessary. Now, admitting that the human race could develop permanent, or at least not easily eradicable, peculiarities, we ask Mr. Gliddon why the red, black, yellow and white varieties of mankind may not have been formed about the year 25,000 before the oldest Egyptian monuments?

Why seek the specific marks of man, in the form of his bones and color of his skin? Evidently his animal nature is the least distinctive part of him. His mind and soul are the grand and glorious peculiarities of his race. These separate him from the brute creation by an immeasurable gulph, while his animal nature almost identifies itself with theirs. In examining claims to manhood we should test them by the intelligence and the passions. Does he think? Does he hope? Does he love? Does he fear? Has he opinions and sympathies? Can he do good and evil? A tear or a smile, or a word; are not these fuller of meaning than the arch of the brow or the swelling of the lip or the hue of the skin? Mr. Laurence once ridiculed the idea of African missions, upon the ground that the formation of the negro skull would be an insuperable barrier to conversion. Nevertheless negroes have been converted with much less effort than was bestowed on Mr. Laurence. Are we to infer then that the insuperable difficulty was in his Caucasian cranium, and that he was an infidel because his head could not contain the dogmas of christianity? If we were to reason as loosely as Mr. Laurence, Gliddon and Nott, have done, we might come to this conclusion, or any other, so only that it should be absurd.

This huge volume will not turn the world upside down. It is by no means a formidable antagonist to christianity. A little knowledge is a dangerous thing, to little people. It produces a swelling about the head, which is too often mistaken for cerebral enlargement, when it is really a mere expansion of brain with diminution of density. Some men have industry and perseverance, and a certain facility in collecting facts; and these make good and useful naturalists, so far as they content themselves with gathering materials for thought. If they attempt to philosophise on their observations, they not uncommonly make a sorry exhibition of their intellectual powers. Unfortunately it is not unusual for men to confound what they know, or think they know, with "science," and claim for it a decisive preponderance in all comparison of opinions. The authors of this work speak as dogmatically and authoritatively about "science," as though it consisted in a very partial knowledge of physiology and history, eked out with the scanty Egyptian pickings of Mr. Gliddon and the magnificent mud rakings of Dr. Dowler. If what these gentlemen have published be "science," it is a very simple thing. Chronology is the result of a sum in multiplication worked out by Dr. Dowler on a few square inches of slate. The most important problem in the natural history of man, is determined by the observation of Jewish looking profiles on an Egyptian monument. The appearance of a negro countenance on a stone, guessed to be 4000 years old, is the "science" before which all the evidences of the christian religion are to go down. The opinions of the ablest geologists, naturalists and archeologists, astronomers, and philosophers are all to be set at naught by the wonderful discoveries of Drs. Nott and Gliddon, who yet have discovered nothing.

Jericho was overthrown by a blast of rams horns, but the manufacturers of this book have essayed to do more with less means.

